# Multiple cryoinjuries modulate the efficiency of zebrafish heart regeneration

**DOI:** 10.1038/s41598-020-68200-1

**Published:** 2020-07-14

**Authors:** Thomas Bise, Pauline Sallin, Catherine Pfefferli, Anna Jaźwińska

**Affiliations:** Department of Biology, University of Fribourg, 1700 Fribourg, Switzerland

**Keywords:** Cell biology, Developmental biology, Cardiology

## Abstract

Zebrafish can regenerate their damaged hearts throughout their lifespan. It is, however, unknown, whether regeneration remains effective when challenged with successive cycles of cardiac damage in the same animals. Here, we assessed ventricular restoration after two, three and six cryoinjuries interspaced by recovery periods. Using transgenic cell-lineage tracing analysis, we demonstrated that the second cryoinjury damages the regenerated area from the preceding injury, validating the experimental approach. We identified that after multiple cryoinjuries, all hearts regrow a thickened myocardium, similarly to hearts after one cryoinjury. However, the efficiency of scar resorption decreased with the number of repeated cryoinjuries. After six cryoinjuries, all examined hearts failed to completely resolve the fibrotic tissue, demonstrating reduced myocardial restoration. This phenotype was associated with enhanced recruitment of neutrophils and decreased cardiomyocyte proliferation and dedifferentiation at the early regenerative phase. Furthermore, we found that each repeated cryoinjury increased the accumulation of collagen at the injury site. Our analysis demonstrates that the cardiac regenerative program can be successfully activated many times, despite a persisting scar in the wounded area. This finding provides a new perspective for regenerative therapies, aiming in stimulation of organ regeneration in the presence of fibrotic tissue in mammalian models and humans.

## Introduction

Unlimited regeneration of damaged organs has fascinated mankind for millennia. This topic was already mentioned in a Greek myth about the punishment of Prometheus, who was sentenced to eternal torment: Every day an eagle came to feed on his liver, and this organ grew back overnight to be eaten again on the next day. The fascination with repeated regeneration occurs through the history of experimental biology. In the eighteenth century, Abraham Trembley (1719–1784) discovered regeneration in the hydra, which can recreate complete structures each time from bisected parts of the body^1^. Now, we know that some species from certain taxonomic groups, such as cnidarians, planarians, annelids and colonial ascidians, can undergo whole body regeneration and show no limitations of regeneration after recurrent fragmentations^2–5^. Among vertebrates, the concept of unlimited organ regeneration has been explored in several species. Experiments with anuran tadpoles have shown that six sequences of limb amputation and regeneration do not interfere with regrowth of a perfect copy of the appendage^6^. A long term study with adult Japanese newts revealed that even aged animals can faithfully regenerate their lens as many as 18 times upon removal^7^. Impressively, the adult caudal fin of zebrafish can efficiently regrow 29 times after amputation^8^. In mammalian models, the ancient legend about Prometheus has been revisited and scientific research provided evidence that rat livers can regenerate even after 12 sequential hepatectomies^9^. However, too many successive regeneration cycles may also give rise to morphological and histological abnormalities.

A recent study on axolotl limb regeneration has brought forth the notion of exhaustion of regenerative capacities^10^. The adult axolotl forelimb can perfectly regenerate only twice. Beyond this, subsequent amputations lead to an increase in anatomical defects of the regenerate and fibrosis. When challenged with five rounds of injuries, more than half of the limbs failed to regenerate. The authors identified that this aberrant regeneration is due to the dysregulation of the EGF family member *amphiregulin,* a factor promoting epidermal thickening^10^. Regenerative limitation has been also shown in the zebrafish retina. After six phototoxic injuries, the regeneration-competent Müller glia cells repeatedly activate the proliferative and morphogenic programs, leading to the restoration of photoreceptors^11^. However, some perturbations have been noted, such as gliosis and cellular hypertrophy, suggesting an imperfect reconstruction of the retina^11^. Another zebrafish organ that displays diminished regenerative response after repeated amputation is the maxillary barbel^12^. These studies suggest that antagonistic signals may accumulate after recurrent injuries, decreasing the efficiency of the subsequent regeneration.

**Figure 1:**
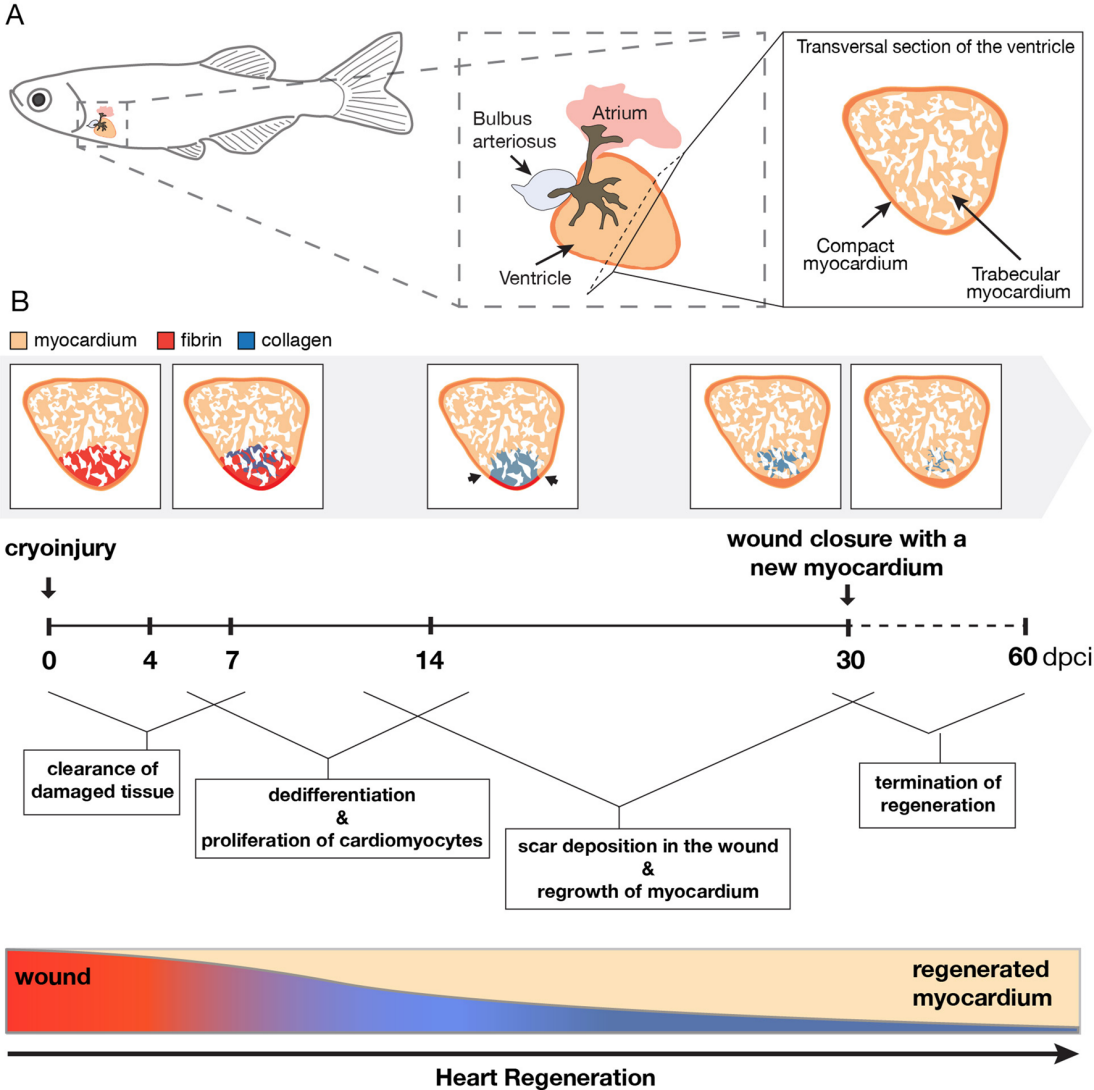
Schematic summary of the regenerative processes after cryoinjury in the zebrafish heart. (**A**) Illustration of the anatomy and histology of an intact zebrafish heart. The ventricle comprises a trabecular myocardium (beige) that is surrounded by a thin layer of a compact myocardium (orange). (**B**) Illustration of the main cellular processes after cryoinjury during the regeneration time. The prominent events are written in the boxes and linked to the specific periods after cryoinjury. The schematic heart sections were based on AFOG histological staining that visualizes the myocardium in beige/orange, collagen in blue and fibrin-like material in red. The injury zone switches from red staining at 4 and 7 dpci, to blue collagen staining after 7 dpci. At the bottom, the rectangular graph depicts a progressive replacement of the wound with a new myocardium. During this time, the wounded tissue undergoes remodeling, starting from the inflammatory state (red gradient) followed by collagenous scar deposition (inverse blue gradient).

Among vertebrates, the zebrafish provides an outstanding model system for heart regeneration. Within 1 month it can substantially restore its damaged ventricle after partial resection, genetic cell ablation or cryoinjury^13–16^. The cryoinjury method mimics a myocardial infarction model, because it involves scarring, which however, is only transient^17–19^. Unlike in mammals, scar deposition in the wounded area is accompanied by cardiomyocyte proliferation in the remaining myocardium. Thus, heart regeneration after cryoinjury relies on the balance between simultaneous reparative and reconstructive processes (Fig. 1). Lineage tracing analyses have provided evidence that the new cardiac tissue derives from proliferative cardiomyocytes at the site of injury^20–23^. Beside the myocardium, regeneration involves the activation of other tissues, such as the epicardium, endocardium, fibroblasts, immune cells, vasculature and nerves and the hormonal system^24–28^. Whether the zebrafish heart can repeatedly deploy this complex regenerative program after multiple injuries remains unknown.

In this study, we sought to determine if adult zebrafish are able to restore their myocardium after several cryoinjuries. We compared the regenerative outcome in four experimental groups that were subjected to one, two, three and six successive cryoinjuries interspaced by 30 days of recovery to achieve recurrent regeneration. The hearts of each group were analyzed at 60 days after the last cryoinjury, to allow for terminal remodeling of the wound (Fig. 1). We based our conclusions on histological and immunofluorescence analysis of various markers in wild type and transgenic fish at several time points during regeneration. Our results demonstrate that the cardiac regenerative capacity is maintained even after six cycles of injury/regeneration. However, we observed impairment of scar resolution, suggesting reduced efficiency in the replacement of fibrotic tissue with a new myocardium.

## Results

### The second cryoinjury damages the regenerated area of the previous cryoinjury

**Figure 2:**
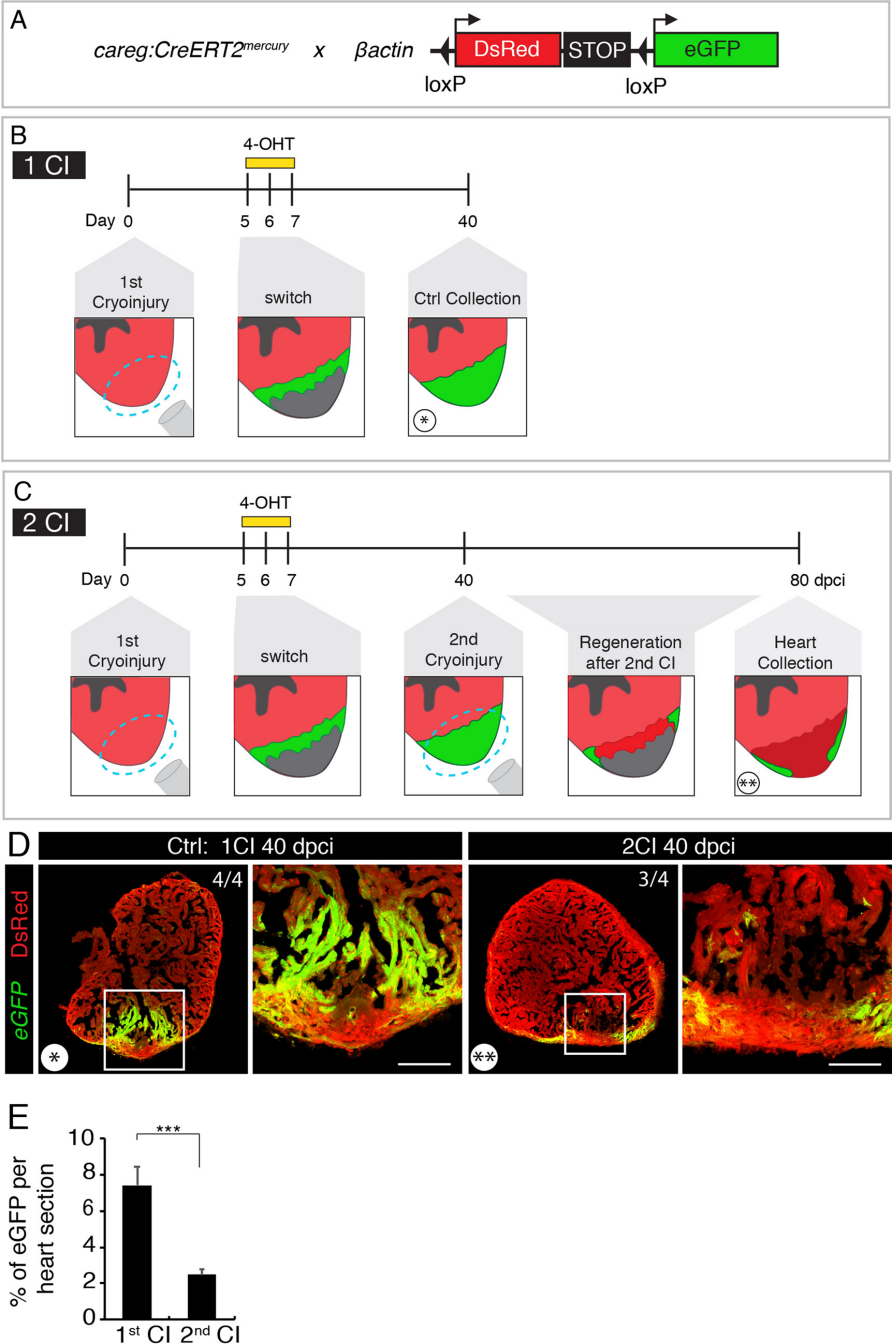
Repeated cryoinjuries target the same part of the zebrafish heart. (**A**) Schematic representation of the transgenic fish lines used for the cell-lineage tracing experiment. (**B**, **C**) Experimental designs. (**B**) The strategy to label the regenerated myocardium after cryoinjury (CI). The entire myocardium expresses *ßactin:DsRed*. *careg:Cre-ERT2* is activated in the peri-injury zone in regenerating cardiomyocytes. Treatment with 4-hydroxytamoxifen (4-OHT) for 2 days starting at 5 dpci (days post-cryoinjury) results in Cre-*loxP* recombination that leads to eGFP expression in the new myocardium, as assessed at 40 dpci. (**C**) The strategy to assess if the regenerated myocardium is damaged by the subsequent cryoinjury. At 40 dpci, another cryoinjury is performed, and hearts are analyzed after subsequent 40 days. (**D**) Cross-sections of zebrafish hearts at 40 dpci after one (*) or two (**) cryoinjuries (CIs). The first regenerated myocardium is labelled by eGFP. The second cryoinjury destroyed this regenerated tissue, as revealed by the loss of the majority of the eGFP-positive myocardium. The number in the upper right corner of each image represents the fraction of analyzed fish with the displayed phenotype. Scale bar = 100 µm. (**E**) Histogram showing the proportion of eGFP-labelled myocardium relative to the ventricular area after one or two cryoinjuries. The 2nd cryoinjury leads to a significant decrease of 4-OHT-induced eGFP expressing cells compared to hearts after one cryoinjury. N = 4. In this and all subsequent figures, frames depict the areas that are shown at higher magnification to the right of each image.

Our experimental approach relies on inducing repeated damage to the same part of the ventricle. Given that the heart does not change its orientation in the body cavity and that the cryoprobe is always inserted from the ventral position, we assumed that the same site of the ventricle would be targeted by each cryoinjury. To test this hypothesis, we used a cell-lineage tracing analysis. Our laboratory has previously identified that the *careg* transgenic reporter is induced in regenerating cardiomyocytes of the peri-injury zone, which gives rise to the new myocardium^22^. To genetically label the regenerated cardiac tissue, we crossed *careg:CreERT2* with ßactin*-loxP-DsRed-STOP-loxP-eGFP* (Fig. 2A). In these fish, the intact myocardium is labelled by *DsRed* expression^20^. At 5 days post cryoinjury (dpci), we exposed the fish to 4-hydroxytamoxifen (4-OHT) for two days to stimulate CreERT2-*loxP* recombination in *careg*-expressing cardiomyocytes (Fig. 2B). At 40 dpci, examination of heart sections revealed that the regenerated myocardium was demarcated by GFP expression, as expected (Fig. 2D). We then carried out an experiment with two cryoinjuries: At 40 dpci (33 days after 4-OHT treatment), we performed another cryoinjury and analyzed the hearts 40 days later (Fig. 2C). We found that most of the hearts (3 hearts out of 4) contained only rudimentary GFP-expression, predominantly in the compact myocardium (Fig. 2D). Quantification of GFP-positive area revealed a threefold reduction of the GFP-labelled myocardium after two cryoinjuries, compared to that after one cryoinjury (Fig. 2E). This finding suggests that the second cryoinjury destroyed most of the previously regenerated ventricle. We concluded that repeated cryoinjuries target the same part of the zebrafish heart, validating our experimental approach.

### Multiple cryoinjuries result in heart regeneration with different degrees of fibrosis

**Figure 3:**
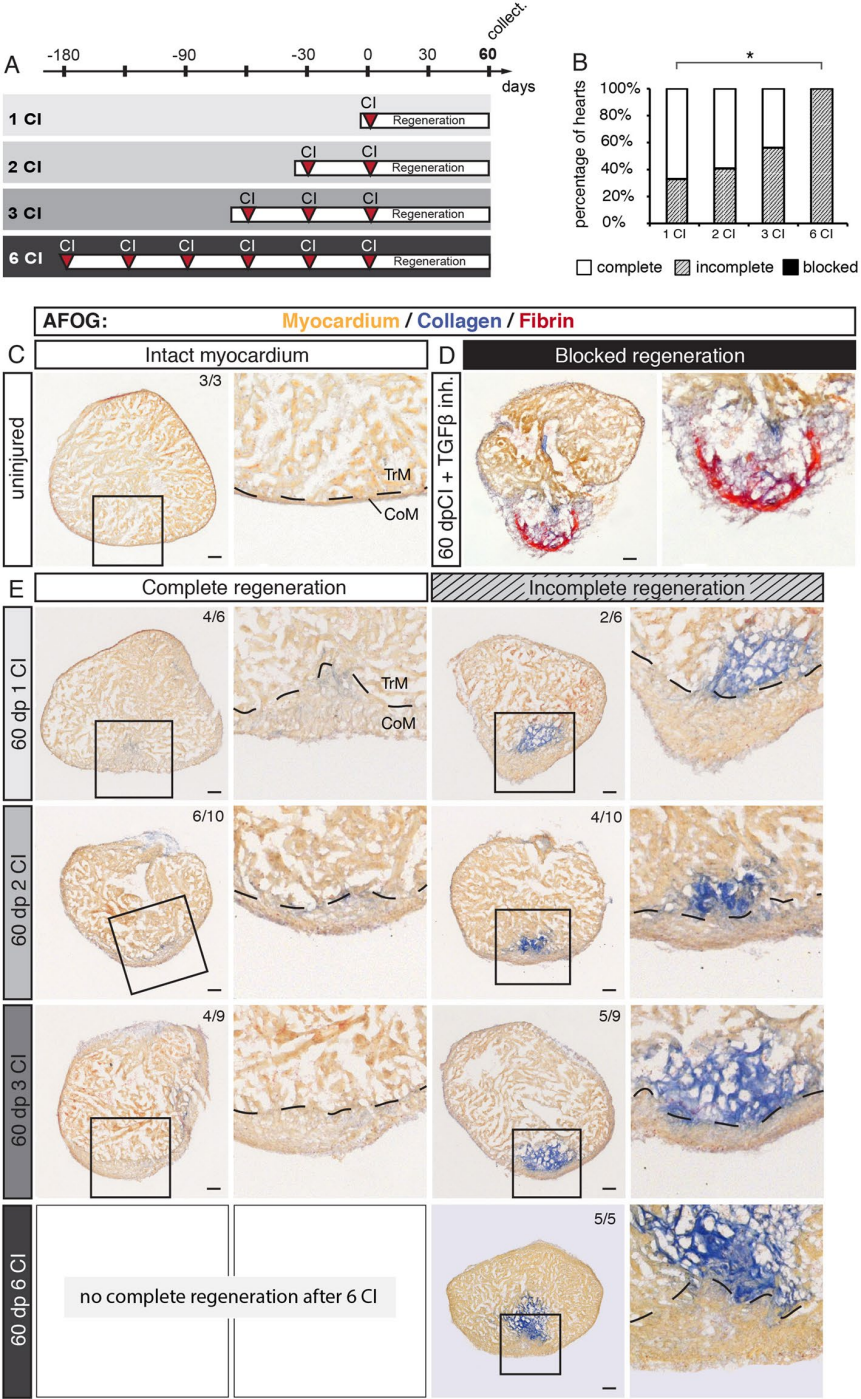
The capacity of complete regeneration is limited after six cryoinjuries. (**A**) Experimental design showing a schedule of cryoinjuries that were performed every month in the same animals. After the last cryoinjury, hearts were allowed to regenerate for 60 days. (**B**) Histograms representing the percentage of zebrafish hearts with complete (white), incomplete (gray) or blocked (black) regeneration after one, two, three or six cryoinjuries (CIs). N ≥ 5. *P < 0.05, Student t-test. (**C**–**E**) Histological staining of cross-sections with AFOG reagent showing the myocardium (beige), fibrin (red) and collagen (blue). (**C**) Uninjured hearts do not contain collagen (blue) in the myocardium. (**D**) An example of blocked regeneration at 60 dpci, following inhibition of the TGF-ß signaling pathway with the chemical antagonist SB431542. Blocked regeneration is featured by the presence of fibrin around the wound, and a lack of myocardium in the wounded area. (**E**) Representative sections of hearts exhibiting complete and incomplete regeneration at 60 dpci after one, two, three or six cryoinjuries. Dashed line in images at higher magnification marks the junctional region between the trabecular (TrM) and compact (CoM) myocardium. The number in the upper right corner of each image represents the fraction of analyzed fish with the displayed phenotype. Scale bar = 50 µm.

Regeneration of the zebrafish heart after cryoinjury is associated with transient fibrosis, which progressively resolves, giving space to a new regrowing myocardium^17–19^. To determine whether the zebrafish heart can regenerate and resorb the scar after several repeated injury/regeneration cycles, we established four experimental groups with one, two, three or six cryoinjuries that were interspaced by 30 days of recovery (Fig. 3A). This time is necessary to close the wound with a new myocardium (Fig. 1B). After the last cryoinjury, we let the fish regenerate during 60 days, in order to reach termination of regeneration^18^. Then, hearts were analyzed using histological staining with the AFOG reagent, which visualizes the intact muscle in orange, collagen in blue and fibrin in red. Ventricles were scored as completely regenerated, when no collagen nor fibrin was visible, like in uninjured control (Fig. 3C). Incomplete regeneration was annotated when a new layer of myocardium covered the wounded area, however, collagenous matrix still persisted in the inner region of the post-injured ventricle (Fig. 3E). In non-regenerated hearts, such as after inhibiting TGF-ß signaling^29^, the wound margin is not covered with a myocardium, but with fibrin or connective tissue (Fig. 3D). Using this classification, we analyzed hearts challenged to undergo multiple regeneration.

Previous studies demonstrated that after ventricular cryoinjury, a majority of fish can fully regenerate the myocardium at 60 dpci, although some fish exhibit incomplete regeneration^30–32^. Consistently, among 6 fish after one cryoinjury, 4 completely regenerated the myocardium (67%), whereas 2 fish displayed incomplete regeneration (33%) (Fig. 3B, E). After two cryoinjuries, 6 fish out of 10 completely regenerated, the other 4, only partially. A similar ratio was present in the group after three cryoinjuries, out of 9 fish, regeneration was complete in 4 animals and incomplete in 5 fish (Fig. 3B, E). However, after six cryoinjuries, all the analyzed ventricles (5 fish) were partially regenerated and none of the animals succeeded to completely resorb the scar (Fig. 3B, E). We concluded that approx. 50% of fish can completely regenerate their ventricles after two and three cryoinjuries, but after six cryoinjuries, the completion of regeneration is impaired or delayed.

The fact that we did not observe a case with a blocked regeneration suggests that the substantial restorative capacity is maintained in all experimental groups. A characteristic feature of the regenerated ventricle is the formation of a new thickened myocardium at the outer margin of the injury zone^22,33^. To determine whether multiple cryoinjuries further affect this phenotype, we measured the thickness of the new compact myocardium. After one cryoinjury, this layer was approx. 5-times thicker than in uninjured ventricles (Suppl. Fig. S1). Interestingly, no additional modulation of this phenotype occurred after two, three or six cryoinjuries. Thus, multiple rounds of regeneration do not further influence myocardial thickening, when compared to one regeneration.

### Multiple cryoinjuries increase accumulation of Collagen XII in the fibrotic tissue

**Figure 4:**
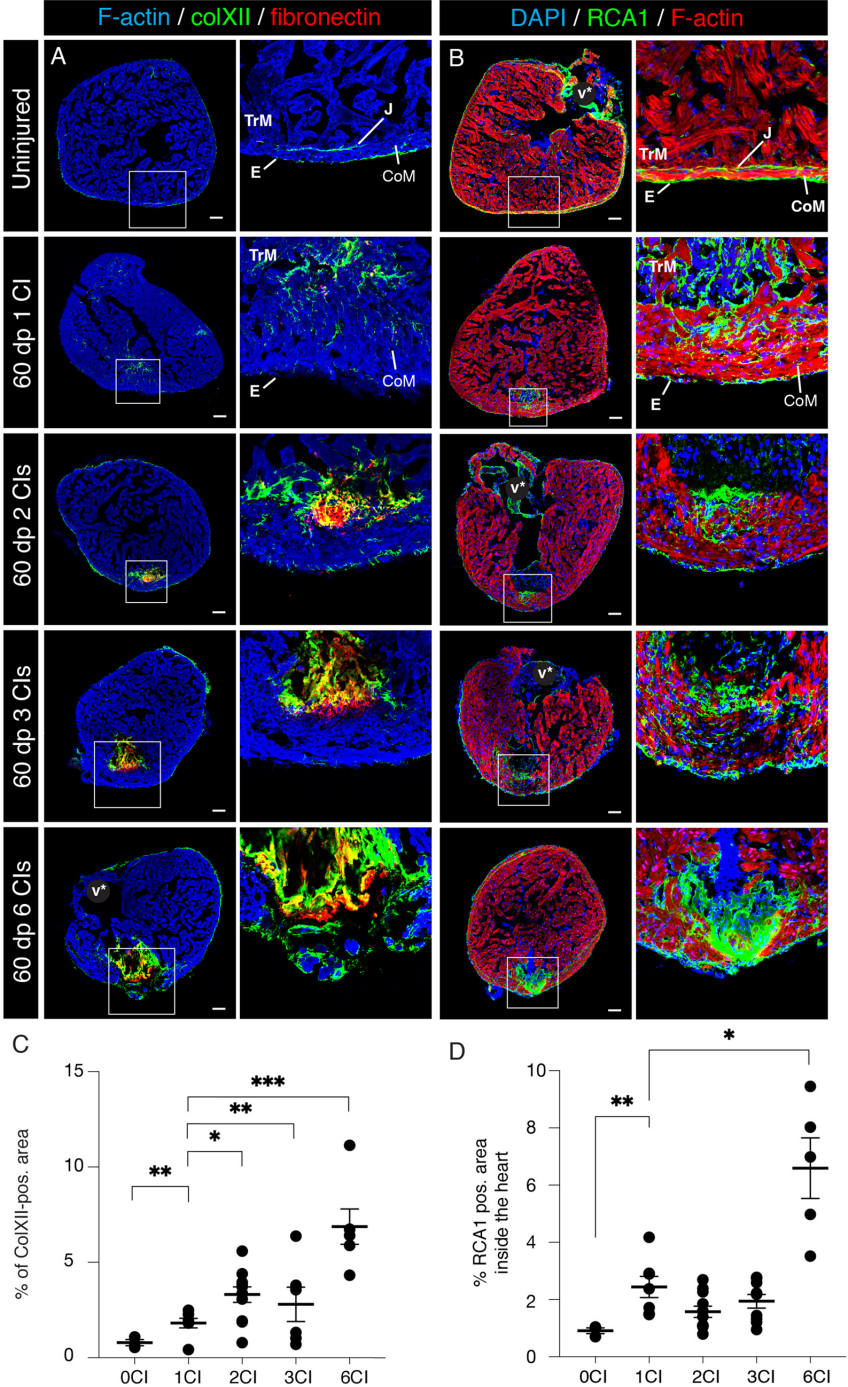
Multiple cryoinjuries enhance deposition of ColXII and connective tissue in the remaining fibrotic tissue. (**A**) Cross sections of adult zebrafish hearts immunostained for the type XII collagen (ColXII; green) and Fibronectin (red) in uninjured hearts and at 60 dpci, following one, two, three or six cryoinjuries. The cardiac muscle is detected by F-actin staining (Phalloidin, blue). In uninjured hearts, ColXII is detected in the epicardium (E) and the junctional region (J) between the compact myocardium (CoM) and the trabecular myocardium (TrM). After cryoinjuries, the fibrotic tissue of partially regenerated hearts contains ColXII and Fibronectin (yellow through an overlay of green and red staining). (**B**) Cross sections of adult zebrafish hearts stained with fluorescein-conjugated lectin *Ricinus communis agglutinin 1* (RCA1, green). The cardiac muscle is detected by F-actin staining (Phalloidin, red). In uninjured hearts, RCA1 labels the valve (V), the epicardium (E) and the junctional region (J) between the compact (CoM) and the trabecular myocardium (TrM). After cryoinjuries, the fibrotic tissue of partially regenerated hearts displays abundant RCA1 labelling. (**C**) Analysis of the percentage of ColXII-positive area per heart section. N ≥ 5. *P < 0.05; **P < 0.01; ***P < 0.001. Scatter plot of the data with a large bar indicating the mean and smaller bars representing the SEM. (**D**) Analysis of the percentage RCA1-positive area per heart section. N ≥ 5. *P < 0.05; **P < 0.01. Scatter plot of the data with a large bar indicating the mean and smaller bars representing the SEM.

The fibrotic tissue of the regenerating heart contains several extracellular proteins, such as tenascin C, fibronectin, fibrillar type I collagen and non-fibrillar type XII collagen (ColXII)^29,34,35^. To determine whether repeated cryoinjuries modulate the composition of the fibrotic tissue, we assessed the distribution of fibronectin and ColXII. Consistent with a previous study^35^, in sections of uninjured ventricles, ColXII was present in the epicardium and in the junctional connective layer between the compact and trabecular myocardium (Fig. 4A). We found that ColXII and fibronectin largely colocalized in the fibrotic tissue of partially regenerated hearts of all experimental groups. Quantification of ColXII per heart section area revealed a progressive increase of this protein, correlating with the number of cryoinjuries, specifically, 2% after one cryoinjury, 3% after two cryoinjuries, 4% after three cryoinjuries and 8% after six cryoinjuries (Fig. 4C). This indicates that repeated cryoinjuries stimulate accumulation of ColXII in the fibrotic tissue.

*Ricinus communis* agglutinin 1 (RCA-1) serves as a marker of tissues lining the compact myocardium, namely the epicardium and the junctional region between the compact and trabecular myocardium^36^. This lectin also labels the valve and the fibrotic tissue of the post-infarcted area (Fig. 4B). Quantification of RCA-1 in the ventricle revealed approx. 3-times more abundant labelling after six cryoinjuries than after one cryoinjury (Fig. 4D). Taken together, we concluded that hearts that underwent six cryoinjuries exhibited an increased fibrosis.

### Characterization of the inflammatory and proliferative phases after multiple cryoinjuries

**Figure 5:**
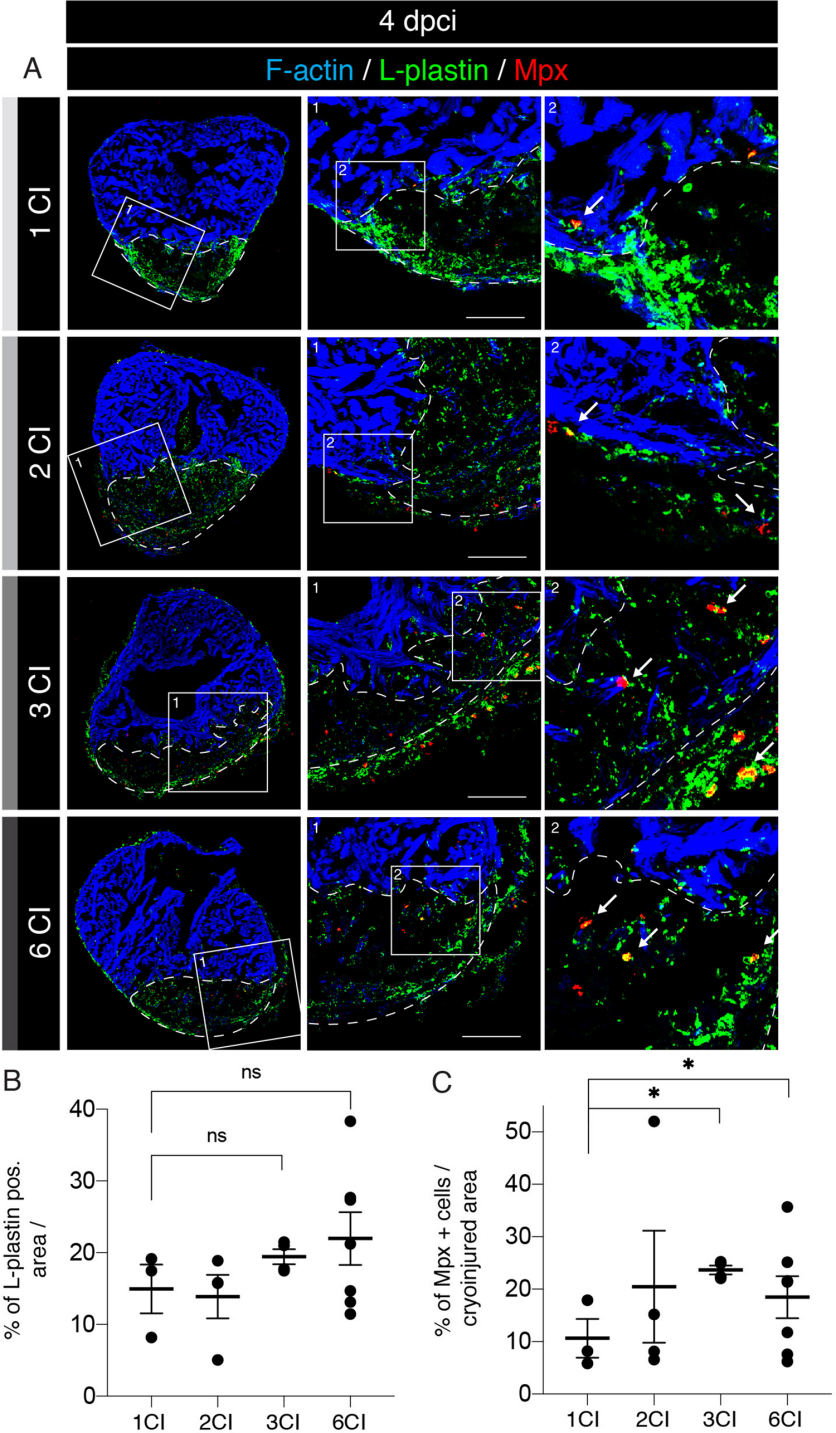
Increased recruitment of Mpx-positive neutrophils at the onset of regeneration after multiple cryoinjuries. (**A**) Cross sections of adult zebrafish hearts immunostained for L-plastin (green) and Myeloperoxydase (Mpx, red) at 4 dpci following one, two three and six cryoinjuries (CIs). The cardiac muscle is detected by F-actin staining (Phalloidin, blue). Both L-plastin and Mpx were detected in the wound. L-plastin/Mpx double positive cells are indicated (white arrow). The dashed line encircles the post-injury zone. Scale bars = 100 µm. (**B**) Analysis of the percentage of L-plastin-positive area within the wound. No significant change (NS) is observed between the experimental groups. N ≥ 3. Scatter plot of the data with a large bar indicating the mean and smaller bars representing the SEM. (**C**) Analysis of the percentage of Mpx-positive area in the wound. Multiple cryoinjuries significantly increase the number of neutrophils compared to specimens after one cryoinjury. N ≥ 3; *P < 0.05. Scatter plot of the data with a large bar indicating the mean and smaller bars representing the SEM.

To understand the effects of multiple cryoinjuries on the heart, we assessed the cellular processes during the initiation of regeneration. The first important step at the beginning of regeneration is the immune response, which is triggered shortly after injury. Phagocytic cells are recruited to the wound for clearance of tissue debris^25,30^. These cells can be detected by immunofluorescence staining against L-plastin, a leukocyte-specific actin-bundling protein and against Myeloperoxidase (Mpx, also abbreviated as Mpo), an enzyme expressed in neutrophils^37,38^. To examine the effect of multiple cryoinjuries on immune cell recruitments, we analyzed hearts at 4 dpci (Fig. 5A). We did not identify any difference in the number of L-plastin positive cells between the experimental groups (Fig. 5B). Only a small increase of Mpx-labelled cells was observed after multiple cryoinjuries, as compared to one cryoinjury (Fig. 5C). Thus, the recruitment of neutrophils seems to be slightly elevated after repeated injuries.

**Figure 6:**
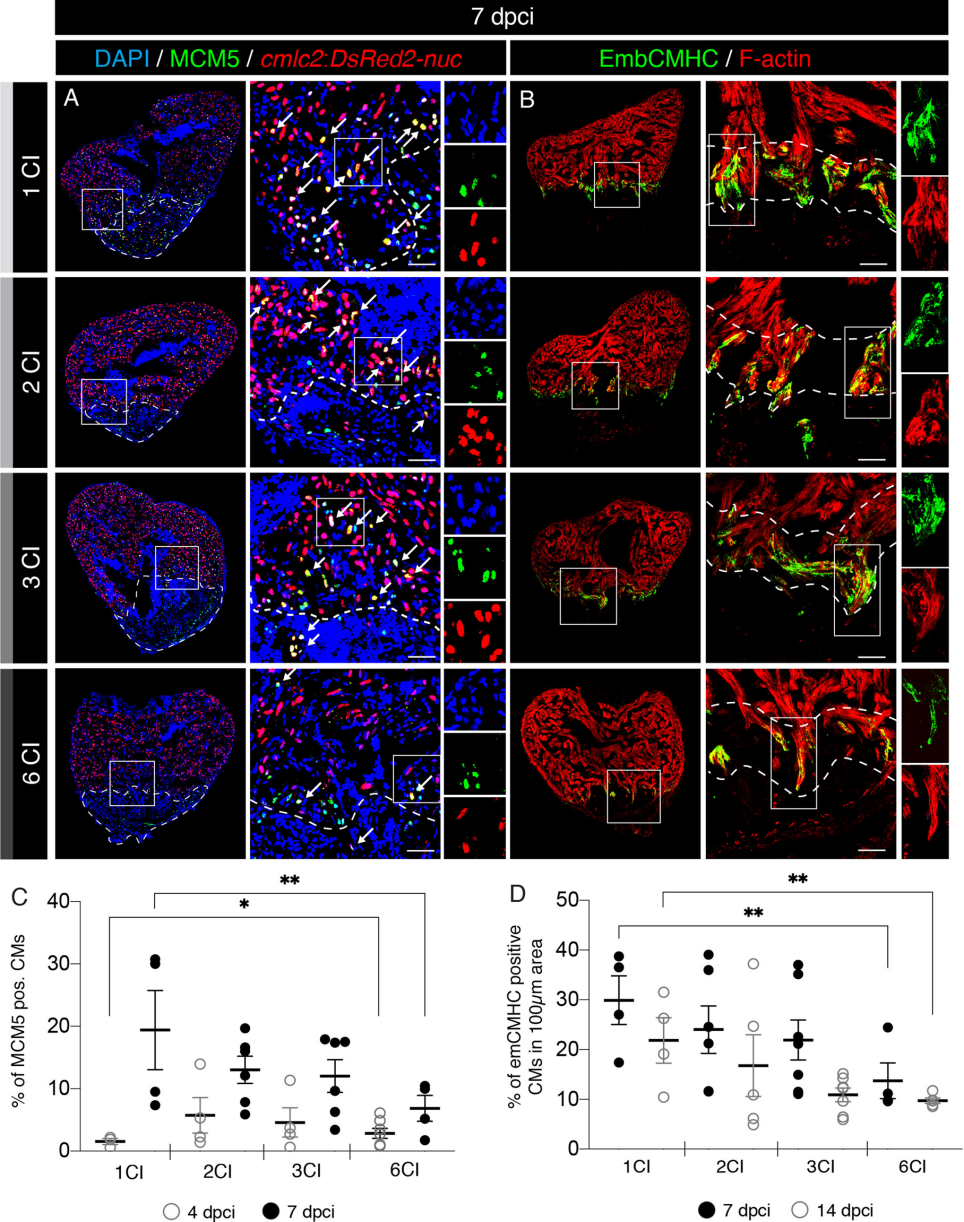
A decreased activation of cardiomyocyte proliferation and dedifferentiation after multiple cryoinjuries. (**A**) Cross sections of transgenic zebrafish hearts at 7 dpci following 1, 2, 3 or 6 CIs expressing nuclear DsRed in cardiomyocytes. Proliferating cells are detected by immunostaining against Minichromosome Maintenance Complex Component 5 (MCM5; green). Proliferating cardiomyocytes are observed (white arrows) by colocalization between *cmlc2:DsRed2-nuc* and MCM5 in the vicinity of the wound (encircled with a dash line). Scale bars = 50 µm. (**B**) Cross sections of adult zebrafish hearts at 7 dpci following 1, 2, 3 or 6 CIs, immunostained for embryonic cardiac myosin heavy chain (EmbCMHC; N2.261; green). The cardiac muscle is detected by F-actin staining (Phalloidin, red). EmbCMHC-positive cardiomyocytes are detected in the peri-injury zone within an area of 100 µm from the injury border (dashed line). (**C**) Analysis of the percentage of MCM5-positive nuclei of cardiomyocytes (CMs) at 4 and 7 dpci, following multiple cryoinjuries. N ≥ 4. *P < 0.05; **P < 0.01. Scatter plot of the data with a large bar indicating the mean and smaller bars representing the SEM. (**D**) Analysis of the percentage of embCMHC-positive cardiomyocytes (CMs) at 7 and 14 dpci, within the peri-injury zone. N ≥ 4. **P < 0.01. Scatter plot of the data with a large bar indicating the mean and smaller bars representing the SEM.

The critical regeneration phase consists of activation of cardiomyocytes, which dedifferentiate and proliferate, mainly at the peri-injury zone. In order to score the effect of multiple cryoinjuries on cardiomyocyte proliferation, we used transgenic fish (*cmlc2:DsRed2-nuc*) that express DsRed2 in cardiac nuclei^39^. We stained sections with an antibody against Minichromosome Maintenance Complex Component 5 (MCM5), a marker of G1/S cell cycle^40^. We found that at 4 dpci, after multiple cryoinjuries, the number of MCM5-positive cardiomyocytes was approx. twice higher than after one cryoinjury (Fig. 6C and Suppl. Fig. S2). This result suggests that at the onset of the regenerative process, cardiomyocytes can enter the cell cycle more rapidly after multiple cryoinjuries. However, this change was only transient, because at 7 dpci, cardiomyocyte proliferation was reduced compared to the samples after one cryoinjury (Fig. 6A, C). This early and short increase of cycling cardiomyocytes at 4 dpci might represent a preconditioning effect. Preconditioning refers to a cytoprotective response that has been imparted by antecedent damage^38,41,42^.

Cardiomyocyte dedifferentiation involves reactivation of cardiac developmental programs, which can be detected by immunostaining with N2.261 antibody that reacts with an embryonic isoform of cardiac myosin heavy chain (embCMHC)^22,43^. To test if the multiple cryoinjuries affect expression of embCMHC, we analyzed hearts at 7 and 14 dpci. We found that this marker was reduced in hearts after six cryoinjuries, suggesting lower dedifferentiation of cardiomyocytes (Fig. 6B,D and Suppl. Fig. S2). We concluded that after six cryoinjuries, the overall activation of cardiomyocytes is decreased, as compared to one injury.

### Dynamics of heart regeneration after multiple cryoinjuries

**Figure 7:**
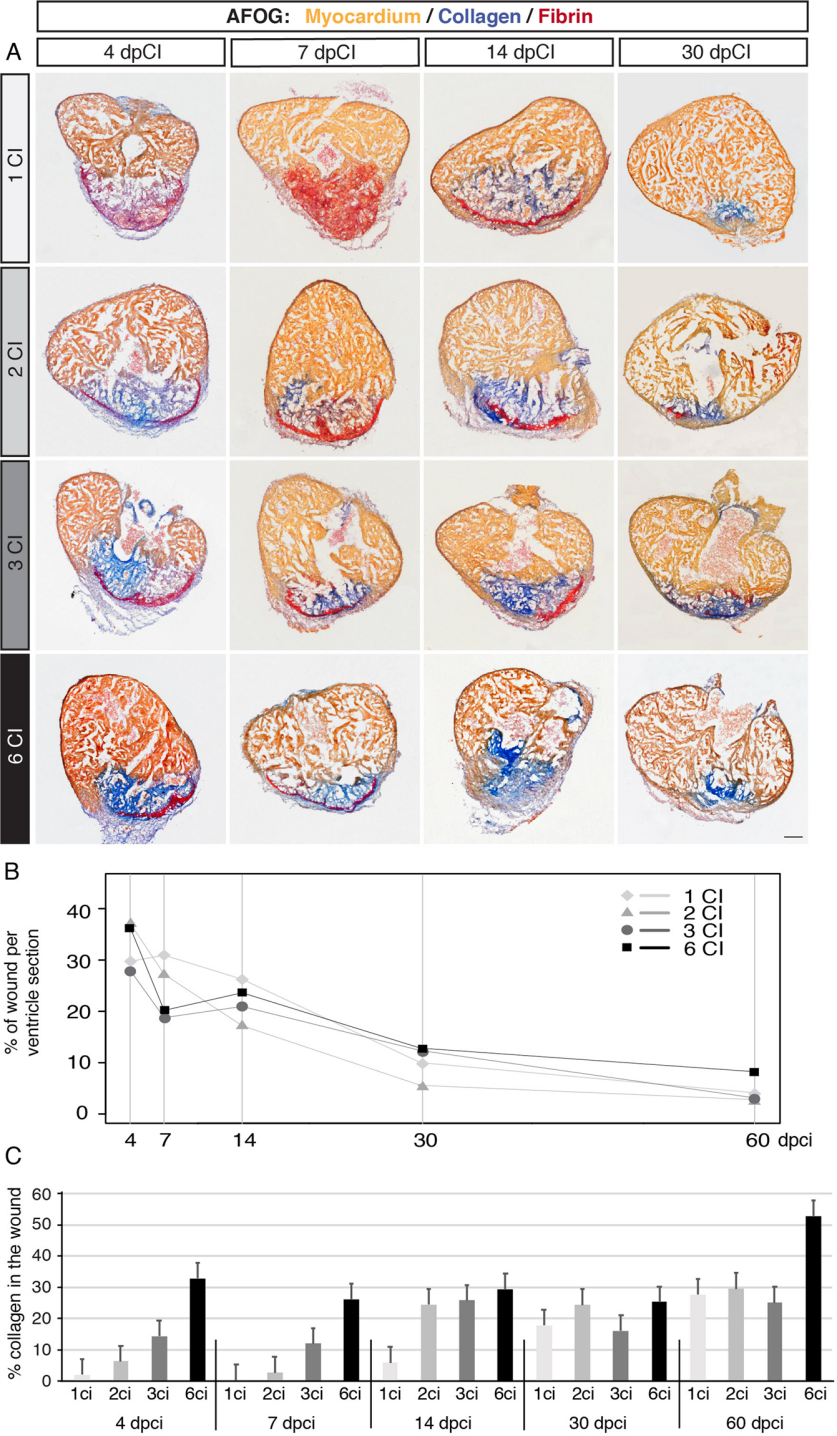
Comparison of regenerative dynamics between hearts after multiple cryoinjuries. (**A**) Representative sections of regenerating hearts stained with the AFOG reagent, showing the myocardium (beige), fibrin (red) and collagen (blue) at different time points following cryoinjuries. At 4 and 7 dpci, the wound contains markedly more collagen after multiple cryoinjuries as compared to that after one cryoinjury. Scale bar = 100 µm. (**B**) Linear representation of the percentage of wounded area per ventricle sections at different regenerative time-points. All experimental groups exhibit similar dynamics of regeneration, but hearts after six cryoinjuries comprise the largest wound as compared to other groups, at 60 dpci. N ≥ 4 hearts, 3 sections per heart. (**C**) Histogram showing the percentage of collagen staining within the remaining wounded area. After multiple cryoinjuries, the amount of collagen is high already at the early time points of regeneration. N ≥ 4 hearts, 3 sections per heart.

The analysis of hearts at 60 dpci revealed reduced efficiency of complete regeneration after 6 cryoinjuries, as compared to one, two or three cryoinjuries (Fig. 3). To characterize the regenerative dynamics for all experimental groups, we performed AFOG staining at intermediate time points, namely at 4, 7, 14 and 30 dpci (Fig. 7A), which corresponds to different phases of regeneration (Fig. 1B). We determined the proportion of injured area within the ventricular surface on three representative sections of all experimental groups (Fig. 7B and Suppl. Fig. S3). Results for 4 and 7 dpci revealed that approx. 20–30% of tissue was damaged in all hearts (Fig. 7B and Suppl. S3A). Little change was observed at 14 dpci. However, at 30 dpci the wound size decreased to approx. 10%, consistent with progressing regeneration. Another month of regeneration resulted in further replacement of the wound, which covered only approx. 5% of ventricular sections in hearts after one, two and three cryoinjuries, and approx. 9% after six cryoinjuries (Fig. 7A,B and Suppl. Fig. S3A). Overall, all experimental groups underwent a similar rate of myocardial regeneration, as shown by ANOVA test of the percentage of wound area per ventricular section at different time points (Fig. 7B and Suppl. Fig. S3B,C).

Although the linear statistical model shows that the dynamics of wound replacement were relatively stable in all groups (Fig. S3B,C), we noticed a difference in the intensity of collagen staining at early time points (Fig. 7A). At 4 and 7 dpci, the wound contained more blue coloration in the post-infarcted tissue after multiple cryoinjuries than after one lesion. Indeed, quantification of the amount of collagen staining per wounded area confirmed this observation. Specifically, at the first week of regeneration, collagen was present in only 1% of wounded area after one cryoinjury, 5% after two cryoinjuries, 15% after three cryoinjuries and 30% after six cryoinjuries (Fig. 7C). At 14 dpci after a single lesion, 5% of fibrotic tissue was filled with collagen fibers, whereas after multiple cryoinjuries, hearts displayed a proportion of collagenous matrix in the wound reaching 25–30% (Fig. 7C). These data indicate that multiple cryoinjuries result in increased fibrosis already at the onset of regeneration. This phenotype can be explained by the persisting remnants of collagen and fibroblasts that were not eliminated from the previous cycle of regeneration^32^. Thus, the repeatedly injured ventricles seem to be conditioned to an early fibrotic response, which however, does not block regeneration.

## Discussion

Certain animals possess an astonishing ability to regenerate their damaged body parts after recurring injuries. However, the reproducibility of the regenerative outcome may vary dependently on the number of lesions, the type of organ and species^10,44^. In this study, we challenged the heart of adult zebrafish with two, three and six cryoinjuries that were separated by a month. First, we used a cell-lineage tracing analysis to demonstrate that a subsequent cryoinjury destroys the previously regenerated myocardium. Then, we analyzed hearts after multiple cryoinjuries at 60 days after the last damage, a time which corresponds to the termination of the restorative process^17–19^. Remarkably, we found that all analyzed specimens displayed at least partial regeneration, defined by the recreation of the outer-most myocardium around the wound. Consistently, cardiomyocytes were able to repeatedly dedifferentiate and proliferate, as assessed during the first two weeks of regeneration. Remarkably, after two and three repeated cryoinjuries, approx. 50% of fish were able to completely resolve the scar. However, after six cryoinjuries, none of the hearts succeeded to fully resorb the scar tissue, showing only incomplete regeneration. These findings indicate that too many lesions can reduce the capacity for efficient replacement of the reparative tissue with a new myocardium.

Collagen is an important component of the post-infarcted myocardium, as it increases tensile strength of the tissue, and provides a substrate for cellular attachment^45^. Our analysis revealed a higher density of collagen in wound tissue, after more regeneration cycles had been induced to the heart. The qualitative increase of fibrosis might act as an obstacle for efficient myocardial replenishment. This conclusion is consistent with the classical hypothesis that the regenerative outcome depends on the interplay between scarring and restoration^10,15,46^. After multiple cryoinjuries, a balance between both processes seems to be shifted towards a reparative scarring response at the expense of the original tissue restitution. Future studies are needed to address this hypothesis, for example, by applying different injury methods, such as genetic cardiomyocyte ablation, which does not cause scarring.

Collagens have been shown to beneficially support the cryoinjured heart^29,32^. Here, we identified that a non-fibrillar type XII collagen (ColXII) increasingly contributed to fibrotic tissue after repeated cryoinjuries (Fig. 4A, C). To our knowledge, this type of collagen has not been reported in scar tissue of the mammalian heart. ColXII is known to form flexible bridges between other fibrillar collagens and other matrix fibers, enhancing chemotactic cellular responses^47^. In zebrafish, this substrate is associated with regeneration of heart, fin and spinal cord, suggesting its stimulating role in the context of disrupted homeostasis^35,38,48–50^. Although fibrosis is considered as detrimental to regeneration, a certain amount of fibrillar collagens and non-fibrillar ColXII might have a beneficial role for cardiomyocyte propagation. The significance of ColXII in the zebrafish heart requires genetic functional analysis.

Although zebrafish can efficiently restore the myocardium, the new tissue comes with some minor abnormalities, as compared to the intact organ. A study using echocardiography has revealed that the regenerated myocardium contracts asynchronously with the remaining heart, even after complete regeneration, suggesting functional imperfectness^51^. Furthermore, the new ventricular area still comprises persisting fibroblasts that have not been fully eliminated, even when no collagenous matrix can be detected in the regenerated ventricle^32^. Whether cardiac fibroblasts become more abundant after repetitive cryoinjuries remains to be further investigated. Interestingly, biomechanical remodeling has been observed after cryoinjury, resulting in a full recovery of normal mechanical stiffness of the regenerated ventricular wall at 35 dpci^52^. At the morphological level, the new compact myocardial layer tends to be thickened, as compared to that of the intact heart^22,33^. This phenotype has been shown to be associated with the impaired restoration of the primordial myocardial layer, which is normally present between the inner trabecular and outer compact myocardium^22^. Interestingly, we found that the width of the compact layer in the regenerate was increased to the similar extent for all hearts, irrespectively of the number of cryoinjuries (Fig. S1). A similar morphological aberration after multiple injuries has also been reported in the zebrafish fin. The caudal fin can repeatedly regrow its shape and size, each time with the same distal shift of the ray branching points, as compared to the original uncut fin^8^. The reproducibility of these abnormalities after multiple rounds of regeneration suggests their functional significance for the respective organ. This phenomenon might indicate that regenerative morphogenesis is driven not only through a sole recapitulation of the stereotypic embryonic programs, but it can be fine-tuned by factors acting in adult organisms. The underlying mechanisms might include anatomical, physiological but also biophysical aspects that modulate the regenerated morphology of the organ.

In summary, our data revealed that even after six cryoinjuries, the zebrafish is able to restore the compact myocardium of the ventricle and substantially replace the wounded area with new cardiac tissue. However, regeneration was incomplete, as the remaining fibrotic tissue contained collagen-rich matrix. This study demonstrates that the presence of fibrosis does not have to preclude a co-existence with the regenerative program. Such a finding opens a new concept for treatments of human organs that are affected by a scar after injury and disease. Despite persisting fibrosis, the therapeutic attempts to reactivate a proliferative capacity in remaining functional cells or cell-based approaches might be promising to achieve at least a partial regeneration. In this context, understanding the interplay between scarring and myocardial replenishment will provide new perspectives in regenerative biology and medicine.

## Methods

### Zebrafish lines and animal use

Wild type AB (Oregon) and transgenic adult zebrafish aged 12–18 months were used in this study. Genetically modified lines were: Tg(*cmlc2:DsRed2-Nuc*)^39^, Tg(*careg:CreERT2*)^*Mercury*^^22^ and Tg(*ßactin-loxP-DsRed-STOP-loxP-eGFP*)^20^. All assays were performed using different animals that were randomly assigned to experimental groups. The exact sample size (N) was described for each experiment in the figure legends and was chosen to ensure the reproducibility of the results. During cryoinjuries and heart collection, fish were anaesthetized with buffered solution of 0.6 mM tricaine (MS-222 ethyl-*m*-aminobenzoate, Sigma-Aldrich) in system water. Animal procedures were approved by the cantonal veterinary office of Fribourg, Switzerland.

### Animal procedures

The TGFβ type I receptor inhibitor SB431542 (Tocris) was dissolved in DMSO at 10 mM stock concentration and was added in fish water for a final concentration of 20 μM. The water with the drug was changed every second day.

For surgeries, anesthetized fish were placed ventral side up in a damp sponge under a stereomicroscope. Chest opening (thoracotomy) was performed by cutting a 1–2 mm incision through the chest with iridectomy scissors (Roboz Surgical Instrument Co.). Ventricular cryoinjuries were performed according to our video protocol^53^. Briefly, the ventricular wall was directly frozen by applying for 23–25 s a stainless steel cryoprobe precooled in liquid nitrogen. To stop the freezing of the heart, room temperature water was dropped on the tip of the cryoprobe, and fish were immediately returned into water.

To collect the heart for fixation, fish were euthanized in 0.6 mM tricaine solution. An incision was made above the heart through the branchial cartilage and the heart was pulled from the body cavity as shown in the video protocol^54^.

### Ethical statement

All animal procedure were conducted in accordance with standard operating procedures at the Department of Biology of the University of Fribourg, Switzerland. Husbandry and animal procedures were approved by the Cantonal Veterinary Office of Fribourg, Switzerland, with the Permit Number 2017_06_FR.

### Histological staining and scoring of regeneration

A triple staining with Aniline blue, acid Fuchsin and Orange-G (AFOG) was performed as previously described in^18^. Briefly, sections were fixed in 10% formalin for 15 min and washed in PBST (PBS + 0.3% Triton-X) for 10 min. The slides were incubated in preheated Bouins fixative (Reactolab, Servion, Switzerland) for 2.5 h at 56 °C and one hour at room temperature. The slides were washed for 20 min in tap water and then transferred into 1% phosphomolybdic acid for 5 min. They were rinsed with distilled water, and incubated for 4 min with AFOG solution (3 g acid Fuchsin, 2 g Orange G, 1 g Anilin Blue, 200 mL acidified distilled water pH 1.1) followed by wash with distilled water. The sections were dehydrated in graded series of ethanol, passed through xylol baths, and mounted with Entellan (107961, Merck Millipore).

Degree of regeneration was scored in a blind-test assessment of AFOG stained sections at 60 dpci. Three sections with evident injury were selected per heart. The criteria for scoring were based on the amount of persisting collagen (blue staining) and the wound closure with a new myocardium. The hearts without persisting collagenous scar were classified as fully regenerated hearts. Hearts with scar remnants that were covered by a new myocardium was considered as incomplete regeneration. The absence of a new myocardium around the wound was considered as a blocked regeneration.

### Immunofluorescence analysis

Immunofluorescence analyses of heart sections were performed as described in^38^. Briefly, hearts were fixed in 2% paraformaldehyde overnight at 4 °C. After wash in PBS, they were equilibrated in 30% sucrose at 4 °C, embedded in tissue freezing media (Tissue-Tek O.C.T.; Sakura) and cryosectioned at a thickness of 16 μm. Slides were stored at − 20 °C. Before staining, slides were transferred to room temperature and the area with sections was encircled with PAP Pen (Vector). Then, the slides were rehydrated in 0.3% Triton-X in PBS (PBST) for 10 min at RT. Blocking solution (5% goat serum in PBST) was applied on the sections for 1 h at RT, then replaced with primary antibody diluted in blocking solution. The incubation was done overnight at 4 °C. After wash in PBST, secondary antibodies diluted in blocking solution were applied on the sections for 2 h at RT. Then, the slides were washed in PBST for 1 h and mounted in mounting medium for fluorescence.

Fluorescein labeled Ricinus Communis Agglutinin I (RCA-1; Vector Laboratories) and Phallodin- Alexa Fluor 568 staining was performed for 30 min at 1:200 in PBS.

The following primary antibodies were used: chicken anti-L-Plastin at 1:1,000 (kindly provided by P. Martin, Bristol)^55^, mouse anti-embCMHC (N2.261) at 1:50 (developed by H.M. Blau, obtained from Developmental Studies Hybridoma Bank), rabbit anti-MCM5 at 1:500 (kindly provided by Soojin Ryu, Heidelberg), rabbit anti-Mpx at 1:500 (GTX128379 GeneTex), guinea pig anti-ColXIIa 1:500 (kindly provided by F. Ruggiero, Lyon). The secondary antibodies (at 1:500) were Alexa conjugated (Jackson ImmunoResearch Laboratories). Phalloidin-Atto-565 (94072, Sigma) and 405 (CruzFluorTM) were used at 1:500. DAPI (Sigma) was applied to label nuclei.

### Image analysis and quantification

Image analysis and quantifications were performed as previously described in^38^. Briefly, after immunofluorescence staining, cardiac tissue imaging was performed at 20 × magnification with confocal microscopes (Leica TCS-SP5 and Leica TCS-SPE-II). N represents the number of fish used in the experiment. Three sections were analyzed per heart. Image analyses were performed using ImageJ 1.49c software. To quantify the number of proliferating cells, the images of the nuclear cell-cycle marker (MCM5) were superimposed with the images of DAPI and *cmlc2:DsRed2-nuc*, which labels cardiac nuclei. The number of proliferating non-CMs was obtained by subtracting the number of MCM5/*cmlc2:DsRed2-nuc*-positive nuclei from the number of MCM5/DAPI-positive nuclei. For quantification of dedifferentiating CMs after cryoinjury, embCMHC positive area was superimposed with the F-actin-positive area within 100 µm from the wound margin. The L-Plastin-positive area was normalized to the total area of the ventricular section. For quantification of specific populations of leucocytes, L-plastin was superimposed with Mpx and normalized to the area of heart sections.

After histological AFOG staining, cardiac tissue imaging was performed at 10 × magnification with a brightfield microscope (Zeiss Axioplan). N represents the number of fish used in the experiment. Three sections were analyzed per heart. Image analyses were performed using ImageJ 1.49c software. The color threshold tool was used to quantify collagen and fibrin levels in ventricle sections.

Concerning all graphical representations, error bars correspond to standard error of the mean (SEM). Significance of differences was calculated using unpaired Student’s *t*-test. Significance between groups were tested using ANOVA test. Statistical analyses were performed with the Microsoft Excel and R software.

## Supplementary information

Supplementary file1 (PDF 18927 kb)

## Data Availability

The authors declare that all data supporting the findings of this study are available within the article and its Supplemental Material files, or from the corresponding author upon request.
