# Supplementary material for: Multiple cryoinjuries modulate the efficiency of zebrafish heart regeneration
Source: Sci Rep. 2020 Jul 14;10:11551. doi: 10.1038/s41598-020-68200-1 (PMC7360767; doi:10.1038/s41598-020-68200-1)

Multiple cryoinjuries modulate the efficiency of zebrafish heart regeneration

Thomas Bise, Pauline Sallin, Catherine Pfefferli and Anna Jaźwińska [1*]

Department of Biology, University of Fribourg, 1700 Fribourg, Switzerland

* corresponding author

Email: anna.jazwinska@unifr.ch

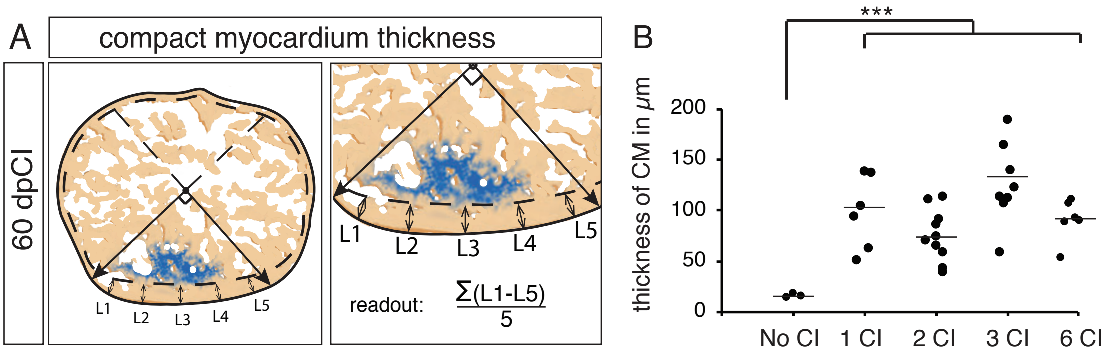

Supp. Figure S1

Multiple cryoinjuries do not modulate the thickness of the new compact myocardium, as compared to one cryoinjury.

(A) Schematic representation of the measurements done on cross sections of hearts at 60 dpci. The average of 5 measurements (L1-5) of the new compact myocardium (CM) was calculated for each heart section. 3 heart sections were assessed for each heart.

(B) Average thickness of the compact myocardium in µm. After single or multiple cryoinjuries, the compact myocardium is thickened to a similar degree, as compared to non-injured hearts. $N \geq 4$ hearts, 3 sections per heart. $^{**}P < 0.01$.

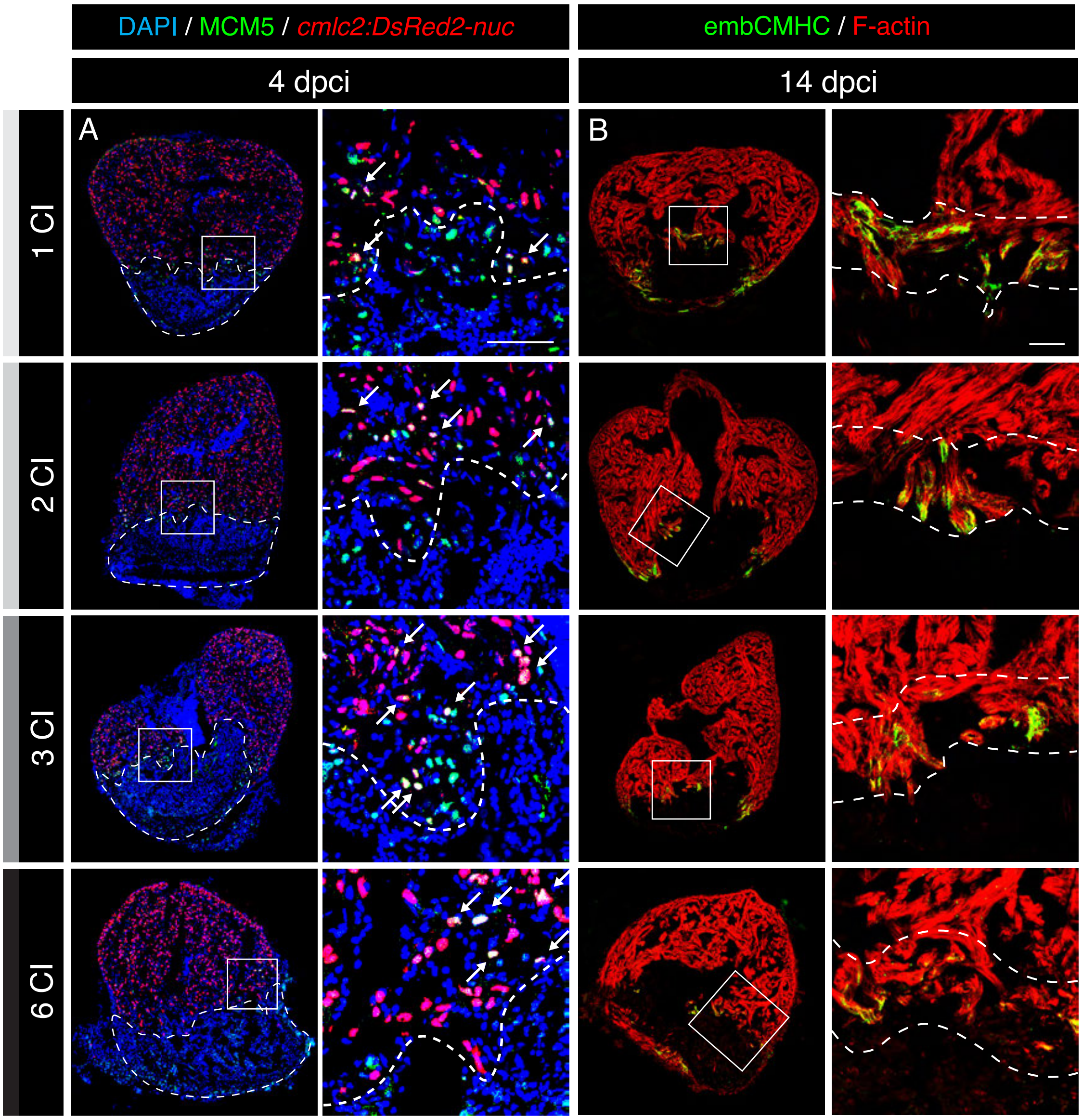

Supp. Figure S2

Effects of multiple cryoinjuries on the cardiomyocyte activation during regeneration.
(A) Cross-sections of transgenic zebrafish hearts at 4 dpci expressing nuclear DsRed in cardiomyocytes. Proliferating cells are detected by immunostaining against Minichromosome Maintenance Complex Component 5 (MCM5; green). Proliferating cardiomyocytes are observed (white arrows) by colocalization between DsRed and MCM5. Scale bars = 50 µm. The dashed line encircles the post-injury zone.
(B) Cross sections of adult zebrafish hearts at 14 dpci after cryoinjuries, immunostained for embryonic cardiac myosin heavy chain (EmbCMHC, N2.261; green). The cardiac muscle is detected by F-actin staining (Phalloidin, red). EmbCMHC-expressing cardiomyocytes are detected in the peri-injury zone within an area of 100 µm from the injury border (dashed line).
Quantifications are shown in figure 6.

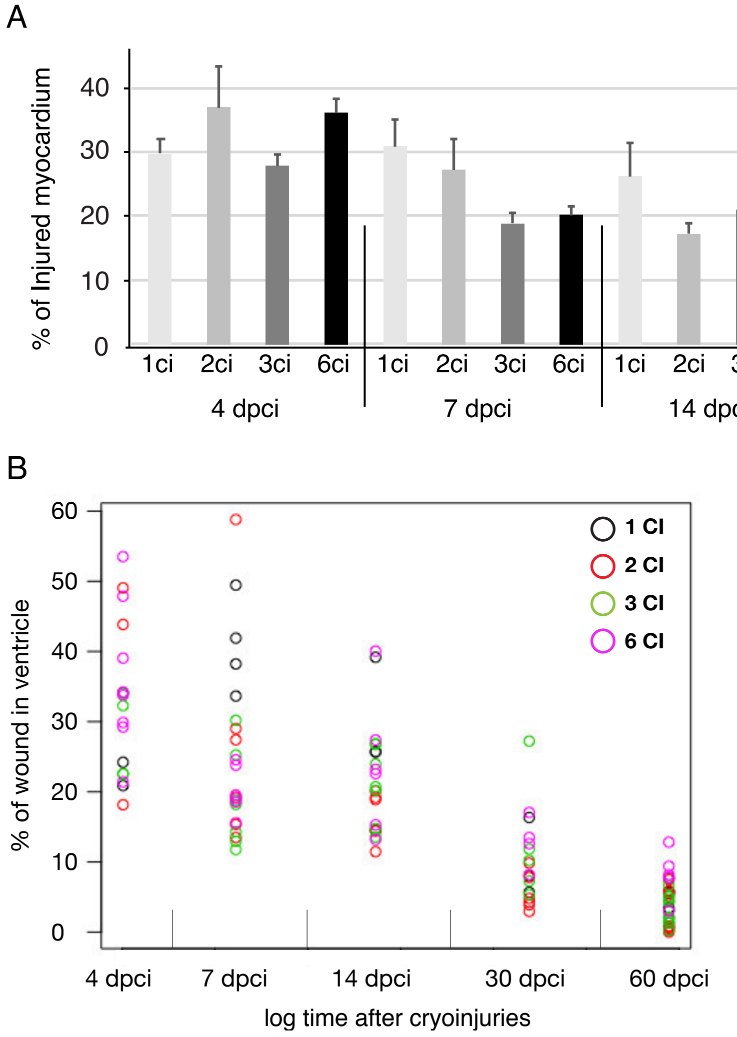

Supp. Figure S3

Comparison of dynamics of heart regeneration between the experimental groups with different numbers of cryoinjuries.
(A) Histogram showing the percentage of the wounded area per ventricular sections, based on histological AFOG staining shown in the main Figure 7. N ≥ 4 hearts, 3 sections per heart.
(B) Scatter plot of the proportion of the wound area per ventricular sections at different regenerative phases (4, 7, 14, 30 and 60 dpci). Note that the time was log10. Different experimental groups (1, 2, 3 and 6 cryoinjuries) are depicted by circles with a distinct color. Each circle corresponds to one heart represented by 3 sections.
(C) Presentation of the data shown in the graph (B) in separate subgraphs for each experimental group (1, 2, 3 and 6 cryoinjuries). Each subgraph shows the regression line which indicates a statistical significant decreasing proportion of the wounded area in ventricular section throughout the time scale. Moreover, an ANOVA analysis indicate no statistical significant difference between those for regression lines (no difference in the intercepts and in the slopes), suggesting a similar dynamics of regeneration. ($p = 0.06947$, N ≥ 4 hearts).

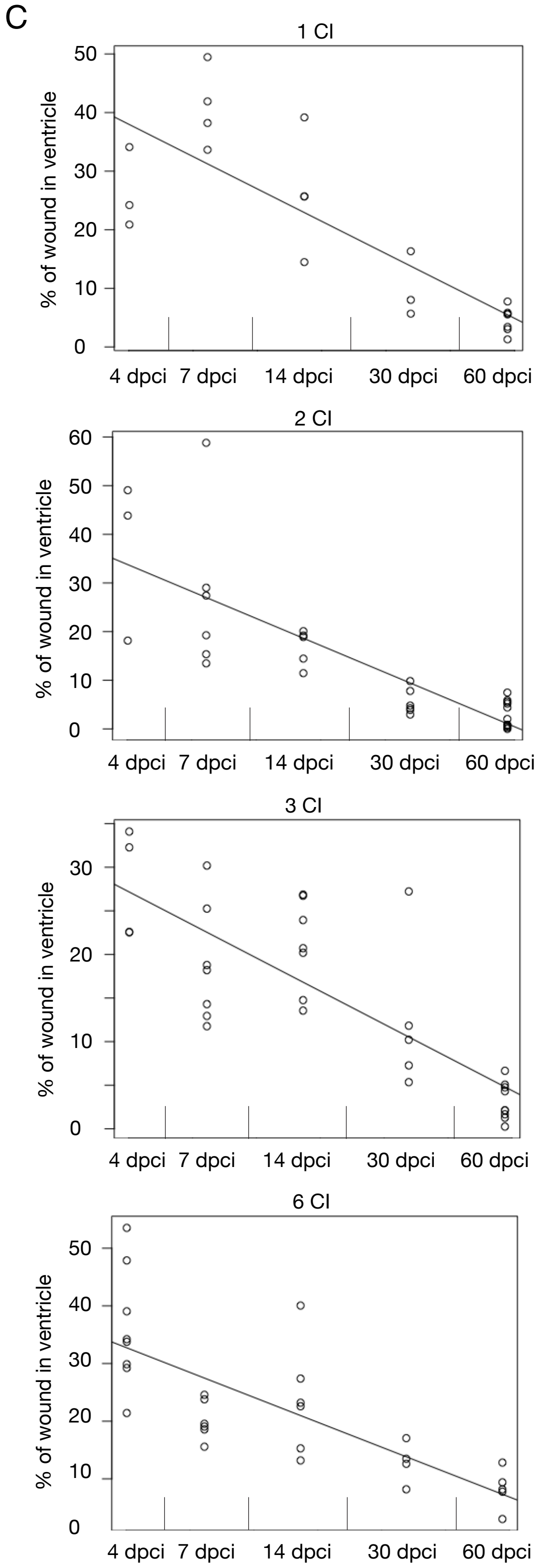

Supplement: Supplementary file 1 — Supplementary file1 (PDF 18927 kb) [file 41598_2020_68200_MOESM1_ESM.pdf]
